# Restitution analysis of alternans using dynamic pacing and its comparison with S1S2 restitution in heptanol-treated, hypokalaemic Langendorff-perfused mouse hearts

**DOI:** 10.3892/br.2016.659

**Published:** 2016-04-19

**Authors:** GARY TSE, SHEUNG TING WONG, VIVIAN TSE, JIE MING YEO

**Affiliations:** 1 School of Biomedical Sciences, Li Ka Shing Faculty of Medicine, University of Hong Kong, Hong Kong, SAR, P.R. China; 2 School of Medicine, Imperial College London, London SW7 2AZ, UK; 3 Department of Physiology, McGill University, Montreal, Quebec H3G 1Y6, Canada

**Keywords:** heptanol, hypokalaemia, S1S2 restitution, dynamic restitution

## Abstract

Action potential duration (APD) and conduction velocity restitution explain the dependence of these parameters on the previous diastolic interval (DI). It is considered to be an adaptive mechanism for preserving diastole at fast heart rates. Hypokalaemia is known to induce ventricular arrhythmias that could be prevented by heptanol, the gap junction uncoupler, mediated through increases in ventricular refractory period (VERP) without alterations in APDs. The present study investigated alternans and restitution properties during normokalaemia, hypokalaemia alone or hypokalaemia with heptanol (0.1 mM) in Langendorff-perfused mouse hearts using a dynamic pacing protocol. APD_90_ alternans were elicited in the epicardium and endocardium during normokalaemia. Hypokalaemia increased the amplitudes of epicardial APD_90_ alternans when basic cycle lengths (BCLs) were ≤65 msec, which was associated with increases in maximum APD_90_ restitution gradients, critical DIs and APD_90_ heterogeneity. Heptanol (0.1 mM) did not exacerbate or reduce the APD_90_ alternans or alter these restitution parameters further. By contrast, endocardial APD_90_ alternans did not show increases in amplitudes during hypokalaemia at short BCLs studied, and restitution parameters were also unchanged. This was true whether in the presence or absence of 0.1 mM heptanol. The study demonstrates that anti-arrhythmic effects of heptanol exerted during hypokalaemia occurred despite exacerbation of APD_90_ alternans. This would suggest that even in the presence of arrhythmogenic APD_90_ alternans, arrhythmias could still be prevented by influencing VERP alone. Restitution data obtained here by dynamic pacing were compared to previous data from S1S2 pacing.

## Introduction

Action potential duration (APD) restitution is the normal shortening of APD that occurs in response to faster heart rates, which is considered to be an adaptive mechanism for preserving diastole at such rates (1). It is defined as the dependence of APD on the previous diastolic interval (DI). Experimentally, APD restitution can be determined using an S1S2 protocol, which gradually shortens the interval between the S1 and S2 stimuli or using a dynamic pacing protocol, which increases the heart rate by progressively reducing the basic cycle length (BCL). Whilst the two methods can be used to measure APD restitution (2), the S1S2 restitution curve is a measure of the immediate response to a change in BCL, whereas the dynamic restitution curve is a measure of the steady-state response (3).

Hypokalaemia is known to exert pro-arrhythmic effects (4), which have been associated with the onset of APD alternans in mouse hearts (5). The latter, in turn, has been attributed to increases in the steepness of APD restitution, critical DIs (5) and maximum APD_90_ reduction (6). Recently, anti-arrhythmic effects of 0.1 and 2 mM heptanol were demonstrated during hypokalaemic conditions (6). In our previous experiments, restitution analysis performed on data obtained during S1S2 pacing confirmed the presence of known abnormalities in APD restitution, in the absence of any changes in conduction velocity (CV) restitution under hypokalaemic conditions. It did not detect any effects of heptanol upon APD or CV restitution. However, this previous study did not explicitly investigate the presence or absence of alternans, which may or may not be due to abnormal APD restitution, nor did it examine restitution parameters by dynamic pacing (6), which is currently the gold standard. Therefore in the present study, the traditional dynamic pacing protocol was used to determine the presence or absence of APD and CV alternans and the effects of heptanol upon these parameters. This permitted their comparisons with values obtained from S1S2 pacing.

## Materials and methods

### 

#### Materials

The present experiments used Krebs-Henseleit solution [119 mM NaCl, 25 mM NaHCO_3_, 4 mM KCl, 1.2 mM KH_2_PO_4_, 1 mM MgCl_2_, 1.8 mM CaCl_2_, 10 mM glucose and 2 mM sodium pyruvate (pH 7.4)] that had been bicarbonate-buffered and bubbled with 95% O_2_ and 5% CO_2_ (7). Hypokalaemic solution was generated by decreasing the amount of KCl added to the Krebs-Henseleit solution to result in a total of 3 mM [K^+^]. Heptanol (Sigma-Aldrich, Dorset, UK; density, 0.82 g ml^−1^) was added directly to the hypokalaemic solution to produce a final concentration of 0.1 mM.

#### Animal model

Wild-type mice (n=13) of the 129 genetic background, between 5 and 7 months of age, were used in the study. These animals were maintained in plastic cages located in the animal facility of the University of Cambridge at 21±1°C with a 12:12-h light:dark cycle and were permitted free access to sterile rodent chow and water at all times. The techniques for the preparation of Langendorff-perfused murine hearts have been fully described previously (6). Mice were sacrificed by cervical dislocation in accordance with Sections 1(c) and 2 of Schedule 1 of the UK Animals (Scientific Procedures) Act 1986. Their hearts were excised and submerged in ice-cold Krebs-Henseleit solution, followed by removal of the surrounding lung tissue. The aorta was cannulated using a tailor-made 21-gauge cannula prefilled with ice-cold buffer, secured using a micro-aneurysm clip (Harvard Apparatus, Kent, UK) and attached to the perfusion system. Retrograde perfusion was started at a rate of 2–2.5 ml min^−1^ using a peristaltic pump (Watson-Marlow Bredel pumps model 505S; Falmouth, Cornwall, UK) with the perfusate successively passing through 200- and 5-µm filters, and heated to 37°C using a water jacket and circulator. The majority of the hearts regained spontaneous contractions and pink colour (~90%). These were subsequently used for experimentation. The remaining hearts were discarded. Perfusion with Krebs-Henseleit solution was maintained for a further 20 min before pharmacological manipulation to minimise residual effects of endogenous catecholamine release.

#### Stimulation protocol

Electrical stimulation of the right ventricular epicardium was carried out immediately following the start of perfusion using paired platinum electrodes (1 mm interpole distance) using regular pacing. This was set at a BCL of 125 msec (8 Hz), which is close to the *in vivo* heart rate of the mouse (8), allowing rate-dependent differences in APDs to be excluded. Square wave pulses of 2-msec duration at a stimulation voltage set to three times the excitation threshold (Grass S48 Stimulator; Grass-Telefactor, Slough, UK) were used, thereby allowing direct comparisons with previous mouse studies of arrhythmogenesis (9–12). The dynamic pacing protocol used to detect alternans involved delivering 100 stimuli at a constant BCL. The BCL was initially set at 180 msec and was decreased by 5 msec every 100 stimuli until a value of 50 msec was reached.

#### Recording parameters

The following parameters were measured: i) APD_90_ alternans, which were calculated by beat-to-beat differences in APD_90_; ii) APD_90_ restitution gradient obtained from restitution curves plotting APD_90_ against the previous DI, assuming its maximum value at the shortest BCL studied; iii) critical DI, DI_crit_, defined as the DI at which the gradient of the APD_90_ restitution curve reaches unity; iv) maximum APD_90_ reduction, a measure of APD_90_ restitution heterogeneity, defined as the maximum APD_90_ reduction observed between the longest and shortest BCL studied; v) CV alternans, which were calculated by beat-to-beat differences in CV; vi) CV restitution gradient obtained from restitution curves plotting CV against the previous DI, assuming its maximal value at the shortest BCL studied; vii) CV restitution curve time constant, τ; viii) maximum CV reduction, a measure of CV restitution heterogeneity, defined as the maximum CV reduction observed between the longest and shortest BCL studied.

Previous studies have associated arrhythmogenesis with three changes in CV restitution: Increased maximum CV restitution gradients, increased time constants of the fitted curves (τ, reflecting reduced CV restitution gradients) (13,14) and increased CV heterogeneity, defined as the maximum CV reduction observed during the dynamic pacing protocol (15). The onset of alternans has previously been attributed to increases in the gradients of restitution curves plotting APD_90_ against the preceding DI and DI_crit_ (5). The present experiments therefore obtained mean values for APD_90_ and DI from all hearts (n=6) at all of the BCLs studied, and fitted them with an exponential function of the form *y* = *y*_0_ + *Ae*^−^x^/^_τ_ by a least squares method using a Levenberg-Marquardt algorithm. *y* and *x* represent mean APD_90_ and mean DI, respectively,

$$\frac{dy}{dx}=\frac{A}{\tau }e^{–x/\tau }$$

whereas *y*_0_, A and τ are constants. The gradient is given by: assuming its maximal value at the shortest BCL studied.

#### Statistical analysis

All values are expressed as the mean ± standard error. Different experimental groups were compared by one-way analysis of variance. P<0.05 was considered to indicate a statistically significant difference. P<0.05 was considered to indicate a statistically significant difference. In figures, *, ** and *** denote P<0.05, 0.01 and 0.001, respectively.

## Results

### 

#### Initial experiments to quantify the APD_90_ alternans

The initial experiments quantified APD_90_ alternans, which have previously been associated with arrhythmogenesis (5), using a dynamic pacing protocol under normokalaemic and hypokaalemic conditions, and hypokalaemia in the presence of 0.1 mM heptanol. This protocol delivered 100 stimuli at a constant BCL, at an initial value of 180 msec and decreased by 5 msec every 100 stimuli until a value of 50 msec was reached. It was not possible to quantify alternans at short BCLs using this protocol in the presence of 2 mM heptanol as ventricular refractory periods (VERPs) increased and CV reduced in a time-dependent manner without reaching a stable value, eventually resulting in conduction block within 4 min of its introduction, preventing a 1:1 stimulus-response. Such effects for 2 mM heptanol on CV and VERP are consistent with those previously reported under normokalaemic conditions (11).

#### Monophasic action potential (MAP) recordings

**Figure 1.**
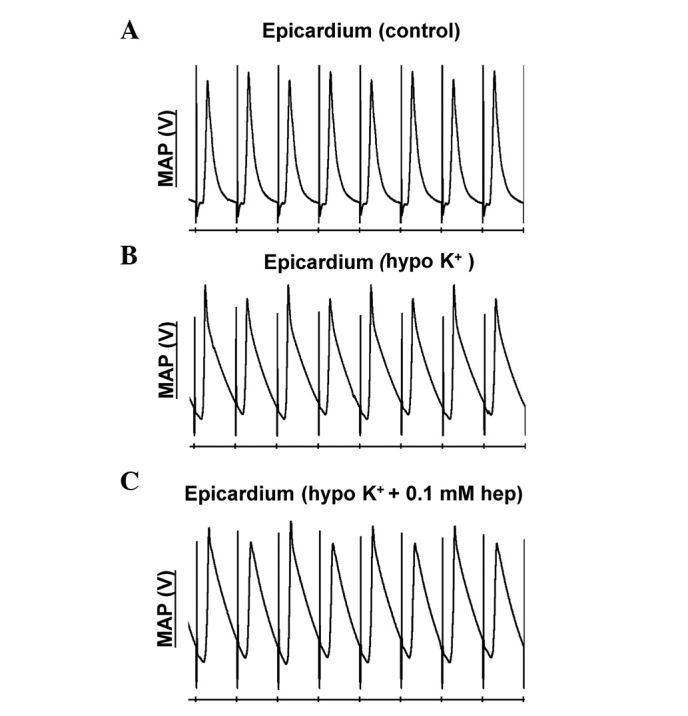
Alternations in conduction velocity and action potential duration observed at a range of basic cycle lengths using a dynamic pacing protocol. MAP recordings obtained from the (A-C) epicardium under control, and hypokalaemic conditions prior and subsequent to the introduction of 0.1 mM heptanol. MAP, monophasic action potential.

**Figure 2.**
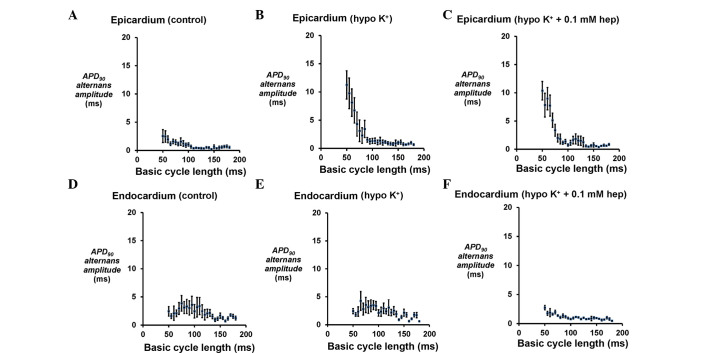
APD_90_ alternans amplitudes. Amplitudes of APD_90_ alternans obtained at a range of BCLs during a dynamic pacing protocol from the (A-C) epicardium under control and hypokalaemic conditions prior and subsequent to the introduction of 0.1 mM heptanol, and from the (D-F) endocardium under the same conditions. The amplitudes of epicardial APD_90_ alternans were significantly increased by hypokalaemia at BCLs ≤65 msec (analysis of variance, P<0.05) and not altered further by 0.1 mM heptanol (P>0.05). By contrast, mp significant difference was observed for those of endocardial APD_90_ alternans between these conditions (P>0.05). APD, action potential duration; BCL, basic cycle length.

Example traces of MAP recordings obtained from the epicardium are shown in Fig. 1 under three pharmacological conditions (n=6). The epicardial (Fig. 2A–C) and endocardial (Fig. 2D–F) APD_90_ alternans increased in magnitude as BCL decreased from 180 to 50 msec under all three cases. However, hypokalaemia significantly increased the alternans at BCLs ≤65 msec in the epicardium (P<0.05); however, no equivalent effects in the endocardium were observed, and none of these values were further altered by 0.1 mM heptanol whether at the epicardium or endocardium (P>0.05).

#### APD restitution curves

**Figure 3.**
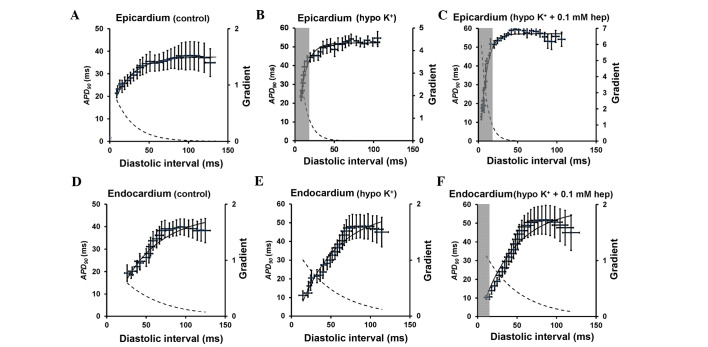
Epicardial and endocardial APD_90_ restitution curves. Restitution curves plotting APD_90_ against the preceding DI obtained from the (A-C) epicardium under control and hypokalaemic conditions prior and subsequent to the introduction of 0.1 mM heptanol and from the (D-F) endocardium under the same conditions. Curves are fitted with mono-exponential growth functions obtained by least-squares fitting to the mean values of APD_90_ and DI (solid lines, left ordinates). Gradients were obtained by differentiation of the fitted functions (broken lines, right axes). The grey boxes indicate the ranges of DI values at which such gradients exceed unity. APD, action potential duration; DI, diastolic interval.

**Figure 4.**
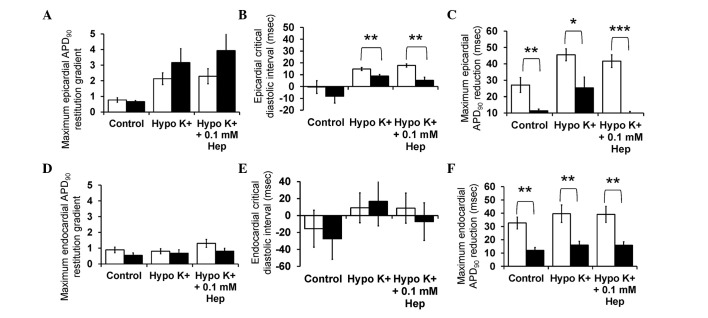
Maximum APD_90_ restitution gradients, critical diastolic intervals and maximum APD_90_ reductions obtained from the (A-C) epicardium and (D-F) endocardium using dynamic pacing (clear bars) and S1S2 pacing (filled bars). APD, action potential duration; Hep, heptanol.

Fig. 3 shows the restitution curves (solid lines, left ordinates) along with their gradients (broken lines, right axes) obtained under control, hypokalaemic conditions before and after introduction of 0.1 mM heptanol from the epicardium (Fig. 3A–C) and endocardium (Fig. 3D–F). The grey boxes indicate ranges of DI values at which such gradients exceed unity (i.e., DIs < DI_crit_). The fitted parameters for the epicardial and endocardial APD restitution curves are detailed in Tables I and II, respectively. APD_90_ decreased as DI decreased under all pharmacological conditions. The maximal APD_90_ restitution gradients, DI_crit_ and maximum APD_90_ reduction at the epicardium (clear bars, Fig. 4A–C) were all significantly increased by hypokalaemia from 0.8±0.2 to 2.1±0.4 (P<0.01), from −0.4±5.4 to 14.9±1.3 msec (P<0.05) and from 27.1±4.5 to 45.5±3.6 msec (P<0.05), respectively. None of these were further altered by 0.1 mM heptanol (P>0.05). By contrast, the maximal restitution gradients, DI_crit_ and maximum APD_90_ reduction at the endocardium (Fig. 4D–F) were not significantly altered by hypokalaemia alone or by 0.1 mM heptanol (P>0.05).

#### CV alternans

**Figure 5.**
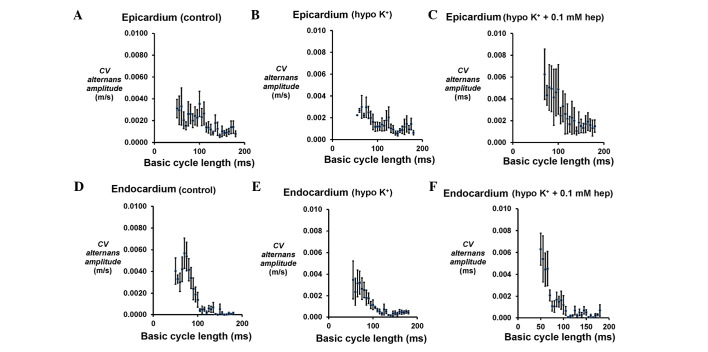
CV alternans amplitudes. Epicardial CV alternans amplitudes obtained at a range of basic cycle lengths during a dynamic pacing protocol obtained from the (A-C) epicardium under control and hypokalaemic conditions prior and subsequent to the introduction of 0.1 mM heptanol, and from the (D-F) endocardium under the same conditions. No significant differences were observed between these conditions (P>0.05). CV, conduction velocity; ms, msec.

The presence of CV alternans would exacerbate any alternation in APD_90_, potentially further increasing arrhythmogenic risk. Therefore, CV alternans were quantified over the same range of BCLs for the epicardium (Fig. 5A–C) and endocardium (Fig. 5D–F). In the epicardium, there was an increase in their magnitudes from 0.0008±0.0003 to 0.0031±0.0008 m/sec (Fig. 5A) as BCL decreased from 180 to 50 msec under the control conditions. However, these values were not altered at any BCL under hypokalaemic conditions alone (Fig. 5B) or following the introduction of 0.1 mM heptanol (Fig. 5C). In the endocardium, CV alternans similarly increased in magnitude with decreasing BCLs over the same range under control conditions (Fig. 5D), and they were not altered further by either hypokalaemia alone (Fig. 5E) or in the presence of 0.1 mM heptanol (Fig. 5F) (P>0.05 in all cases).

#### CV restitution curves

**Figure 6.**
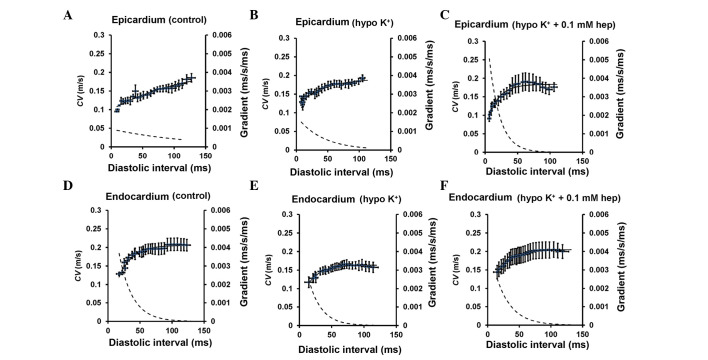
CV restitution curves. Restitution curves plotting CV against the preceding DI obtained from the (A-C) epicardium under control and hypokalaemic conditions prior and subsequent to the introduction of 0.1 mM heptanol, and from the (D-F) endocardium under the same conditions. Curves are fitted with mono-exponential growth functions obtained by least-squares fitting to the mean values of CV and DI (solid lines, left ordinates). Gradients were obtained by differentiation of the fitted functions (broken lines, right axes). CV, conduction velocity; DI, diastolic interval; ms, msec.

**Figure 7.**
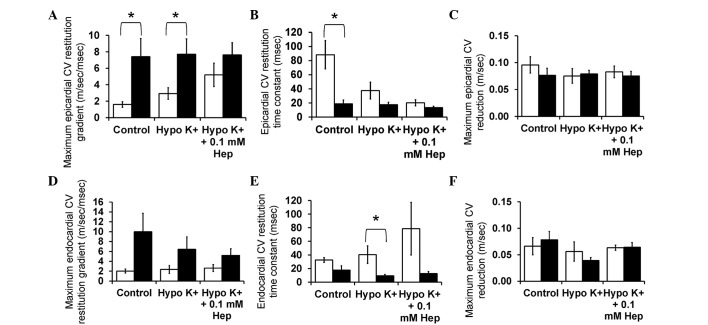
Maximum CV restitution gradients, time constants of restitution curves and maximum CV reductions from the (A-C) epicardium and (D-F) endocardium using dynamic pacing (clear bars) and S1S2 pacing (filled bars). CV, conduction velocity; Hep, heptanol.

Fig. 6 shows CV restitution curves (solid lines, left ordinates) obtained under control and hypokalaemic conditions before and after introduction of 0.1 mM heptanol from the epicardium (Fig. 6A–C) and endocardium (Fig. 6D–F). The fitted parameters are summarized in Tables III and IV. CV decreased as DI decreased under all pharmacological conditions. In the epicardium, hypokalaemia alone did not appear to significantly influence either maximum CV restitution gradients (Fig. 7A) or the time constant τ (Fig. 7B) (P>0.05). By contrast, hypokalaemia with 0.1 mM heptanol increased the maximum CV restitution gradients from 1.6±0.3 to 5.2±1.4 (P<0.05) and decreased τ from 88.3±20.2 to 20.2±4.37 msec (P<0.05). The maximum CV reductions were not significantly different between the three pharmacological conditions (Fig. 7C) (P>0.05). In the endocardium, no difference was observed in any of the CV restitution parameters between these pharmacological conditions (Fig. 7D–F; P>0.05).

Finally, it was possible to compare the restitution parameters obtained in this study using the dynamic pacing protocol with those previously obtained during S1S2 pacing. The latter results are reproduced in the present study (with permission) and represented by filled bars in Fig. 4 (APD data) and Fig. 7 (CV data). Significant differences in values obtained during dynamic pacing and S1S2 pacing data are denoted by asterisks. No difference was observed for APD_90_ restitution gradients obtained by dynamic pacing and S1S2 pacing whether in the epicardium or endocardium under the three pharmacological conditions (P>0.05). By contrast, epicardial DI_crit_ values obtained from dynamic pacing were significantly larger than those obtained during S1S2 pacing during hypokalaemia alone and in the presence of 0.1 mM heptanol (P<0.01). Endocardial DI_crit_ values were not significantly different by the pacing method under the three pharmacological conditions studied. Notably, maximum APD_90_ reduction was significantly larger when dynamic pacing was used whether in the epicardium (Fig. 4C) or endocardium (Fig. 4F) for all pharmacological conditions studied. For CV restitution data, maximum CV restitution gradients in the epicardium were larger when obtained using S1S2 pacing compared to dynamic pacing for control and hypokalaemia alone (P<0.05), but not for hypokalaemia in the presence of 0.1 mM heptanol (P>0.05). This is reflected by shorter time constants τ using S1S2 pacing. As opposed to the maximum APD reduction, maximum CV reduction in either the epicardium or endocardium did not vary between the two pacing methods under the three pharmacological conditions (P>0.05).

In summary, all the aforementioned findings demonstrate that hypokalaemia increased the magnitudes of epicardial APD_90_ alternans at short DIs associated with increased restitution gradients, DI_crit_ and APD heterogeneity, without effects on CV restitution. Heptanol at 0.1 mM did not further influence APD or CV restitution. The dynamic pacing and S1S2 pacing are equally effective for eliciting abnormal restitution.

## Discussion

Abnormal electrical restitution predisposes to ventricular arrhythmogenesis through the production of arrhythmogenic alternans. Different protocols can be used to examine different aspects of restitution: S1S2 pacing can be used to measure the immediate response of cardiac tissue to a change in the BCL, whereas dynamic pacing can be used to determine the steady-state response. In the present study, APD and CV restitution was examined in normokalaemic and hypokalaemic conditions alone and hypokalaemia in the presence of 0.1 mM heptanol, a gap junction inhibitor, using a dynamic pacing protocol. Subsequently, this permitted comparisons with previously published results on S1S2 restitution under similar experimental conditions (6).

Experiments using dynamic pacing protocol showed increasing amplitudes of epicardial and endocardial APD_90_ alternans with progressive shortenings in BCLs under control and hypokalaemic conditions prior and subsequent to the introduction of 0.1 mM heptanol. Hypokalaemia significantly increased the amplitudes of APD_90_ alternans at BCLs ≤65 msec in the epicardium but not the endocardium, in agreement with previous findings (5). These were not further altered by 0.1 mM heptanol, suggesting that it was able to exert anti-arrhythmic effects despite the persistence of arrhythmogenic APD alternans. In the present study, it was not possible to investigate alternans or restitution properties using the dynamic pacing protocol during exposure to a higher concentration of heptanol (2 mM) due to its actions in increasing VERPs and producing conduction block within short periods of time, resulting in the breakdown of the 1:1 stimulus-response. This is consistent with its previously reported inhibitory effects on sodium channels at 2 mM resulting in progressive reductions in CV and ultimately conduction block (16). However, it appeared to be possible to investigate the effects of 2 mM heptanol previously using S1S2 pacing as this protocol only lasts <120 sec (6), whereas the dynamic pacing protocol used in our study lasted ≥5 min.

Restitution analysis demonstrated hypokalaemia increased the maximum APD_90_ restitution slopes and DI_crit_ in the epicardium but not in the endocardium, in agreement with previous findings (5). By contrast, the arrhythmogenesis observed in guinea pig hearts during hypokalaemia was also associated with APD_90_ alternans but this was despite restitution gradients becoming less steep (17). Notably, in guinea pig hearts, endocardial APD_90_ restitution gradients were also increased by hypokalaemia (17), as opposed to in canine (18) or mouse hearts in which they were unaffected (5,6). APD_90_ restitution gradients and DI_crit_ were not further altered by 0.1 mM heptanol.

The presence of CV alternans would exacerbate any alternation in APD_90_, potentially further increasing arrhythmogenic risk, by converting spatially concordant alternans to spatially discordant alternans. CV alternans increased in magnitude with decreasing BCLs decreased but these were not further exacerbated by hypokalaemia alone, nor further altered by 0.1 mM heptanol. Furthermore, alterations in CV restitution gradients, time constants of fitted restitution curves or increased CV heterogeneity (defined as maximum CV reduction at the longest and shortest BCL studied) may predispose to arrhythmias (14,15). Restitution analysis demonstrated increased CV restitution gradients and decreased without altering either CV alternans amplitudes or CV heterogeneity during hypokalaemia. No further changes were observed following 0.1 mM heptanol treatment. These results complement the previous findings associating arrhythmogenesis with increased CV alternans amplitudes and reduced CV restitution slopes using theoretical ion channel models (13) and experimentally using the electromechanical uncoupling drug diacetyl monoxime (14). By contrast, the arrhythmias induced by the electromechanical uncoupling drug methoxyverapamil (D600) in rabbit hearts were associated with increased CV heterogeneity (15).

The present experiments demonstrate that, in general, APD and CV restitution gradients obtained during dynamic pacing are in agreement with those obtained previously using an S1S2 protocol (5,6), with two differences observed between the two methods. Firstly, the present study found that maximum APD_90_ reduction was larger for dynamic pacing compared to for S1S2 pacing. By contrast, in guinea pig hearts, APD restitution gradients obtained using the S1S2 method were larger than those obtained during dynamic pacing (19). In the present study, the apparent lack of difference may be due to the low statistical power to detect differences as a small number of hearts was used. Secondly, dynamic pacing revealed increased CV restitution gradients in the presence of 0.1 mM heptanol during hypokalaemia, a finding that was not apparent using S1S2 pacing (6).

In conclusion, the present study compared results of restitution analysis using the dynamic pacing technique with those obtained previously using a S1S2 protocol. It found that restitution data obtained by the two methods are largely agreeable. This would support the use of S1S2 protocol for examining restitution and justifying its use in experimental and clinical settings, which was previously discussed (19). Certain caution must be taken when interpreting restitution data obtained from S1S2 pacing, as a dynamic pacing protocol was shown to be more representative of the APD dynamics observed at high heart rates (20,21). Although it is the gold standard, dynamic pacing is often avoided in clinical situations as regular pacing at high heart rates can induce myocardial ischaemia (22,23). The advantage of the PES protocol is therefore safety in a clinical setting, as this does not rely on tachycardia pacing (19,21). Using such a protocol, abnormal electrical restitution, which can predispose to the development of arrhythmogenic alternans, can be determined (24).

## Figures and Tables

**Table I.** Parameters for epicardial APD_90_ restitution curves obtained during dynamic pacing. APD, action potential duration.

Condition	y_o_, msec	A, msec	τ, msec
Control	39.5±6.4	−30.3±6.2	25.0±5.6
Hypo K^+^	53.2±2.4	−50.5±4.7	14.6±3.8
Hypo K^+^+0.1 mM heptanol	57.6±2.2	−59.4±2.5	10.4±1.4

APD, action potential duration.

**Table II.** Parameters for endocardial APD_90_ restitution curves obtained during dynamic pacing. APD, action potential duration.

Condition	y_o_, msec	A, msec	τ, msec
Control	47.2±4.8	−59.1±11.5	55.0±15.2
Hypo K^+^	60.8±21.2	−60.2±21.4	53.2±26.1
Hypo K^+^+0.1 mM heptanol	83.5±27.4	−87.3±28.2	64.9±18.5

APD, action potential duration.

**Table III.** Parameters for epicardial conduction velocity restitution curves obtained during dynamic pacing.

Condition	y_o_, m/sec	A, m/sec	τ, msec
Control	0.29±0.09	−0.22±0.089	68.3±6.9
Hypo K^+^	0.20±0.01	−0.11±0.019	37.5±12.0
Hypo K^+^+0.1 mM heptanol	0.18±0.02	−0.12±0.031	20.2±4.4

**Table IV.** Parameters for endocardial conduction velocity restitution curves obtained during dynamic pacing.

Condition	y_o_, m/sec	A, m/sec	τ, msec
Control	0.22±0.02	−0.13±0.030	34.6±3.5
Hypo K^+^	0.17±0.01	−0.09±0.029	40.4±12.8
Hypo K^+^+0.1 mM heptanol	0.27±0.06	−0.18±0.053	78.6±38.7
